# Cattle feeding tendency of *Anopheles* mosquitoes and their infection rates in Aradum village, North Wollo, Ethiopia: an implication for animal-based malaria control strategies

**DOI:** 10.1186/s12936-023-04516-3

**Published:** 2023-03-07

**Authors:** Tsegaye Eshetu, Nigatu Eligo, Fekadu Massebo

**Affiliations:** Department of Biology, College of Natural Sciences, Arba Minch, Ethiopia

**Keywords:** Blood meal sources, Pit shelter, Clay pot, PSC, Aradum, Wollo

## Abstract

**Background:**

Surveillance of indoor and outdoor resting malaria vector populations is crucial to monitor possible changes in vector resting and feeding behaviours. This study was conducted to assess the resting behaviour, blood meal sources and circumsporozoite (CSP) rates of *Anopheles* mosquito in Aradum village, Northern Ethiopia.

**Methods:**

Mosquito collection was conducted from September 2019 to February 2020 using clay pots (indoor and outdoor), pit shelter and pyrethrum spray catches (PSC). The species of *Anopheles gambiae* complex and *Anopheles funestus* group were identified using polymerase chain reaction (PCR). Enzyme-linked immunosorbent assay (ELISA) was done to determine CSP and blood meal sources of malaria vectors.

**Results:**

A total of 775 female *Anopheles* mosquitoes were collected using the clay pot, PSC and pit shelter. Seven *Anopheles* mosquito species were identified morphologically, of which *Anopheles demeilloni* (593; 76.5%) was the dominant species followed by *An. funestus* group (73; 9.4%). Seventy-three *An. funestus* group screened by PCR, 91.8% (67/73) were identified as *Anopheles leesoni* and only 2.7% (2/73) were found to be *Anopheles parensis.* The molecular speciation of 71 *An. gambiae* complex confirmed 91.5% (65/71) of *Anopheles arabiensis*. The majority of *Anopheles* mosquitoes were collected from outdoor pit shelter (42.2%) followed by outdoor clay pots. The majority of the blood meal of *An. demeilloni* (57.5%; 161/280), *An. funestus *sensu lato 10 (43.5%) and *An. gambiae* (33.3%; 14/42) originated from bovine. None of the 364 *Anopheles* mosquitoes tested for *Plasmodium falciparum* and *Plasmodium vivax* sporozoite infections were positive.

**Conclusion:**

Since the *Anopheles* mosquitoes in the area prefer to bite cattle, it may be best to target them with an animal-based intervention. Clay pots could be an alternative tool for outdoor monitoring of malaria vectors in areas where pit shelter construction is not possible.

## Background

Malaria is a major health problem globally and, according to the latest world malaria report, there were an estimated 247 million malaria cases and 619,000 malaria deaths worldwide [1]. Africa has the greatest burden of malaria, with 95% of malaria cases and 96% of deaths [1]. The majority (85%) of deaths took place among those under five years of age. *Plasmodium falciparum* is the most fatal and widespread malaria parasite in the African continent [1]. Unlike several African countries, malaria is extremely unstable and seasonal in Ethiopia [2]. The primary vector is *Anopheles arabiensis,* while secondary vectors, such as *Anopheles pharoensis, Anopheles funestus* and *Anopheles nili,* play a minor role [2]. Current malaria vector control tools are indoor residual spraying (IRS), insecticide-treated nets (ITNs) [2], and larval source management (LSM) as a supplementary tool [3]. Intensive use of these insecticide-based interventions may encourage malaria vectors to develop resistance [4]. In addition, such interventions may affect the resting and feeding habits of *Anopheles* mosquitoes [5]. Some mosquitoes may switch to feeding on animals, which can affect the effectiveness of interventions [6] because they may rest outdoors to avoid exposure to indoor chemicals [5]. Malaria mosquito surveillance and monitoring are, therefore, key to understanding their response to interventions.

Human Landing Catch (HLC) has been considered the gold standard method for sampling malaria mosquitoes [7]. However, it can expose collectors to infectious mosquitoes and finding an alternative method can be important. Pyrethrum spray catches (PSC) and pit shelter collection techniques are commonly used for indoor and outdoor resting mosquito sampling, respectively [8], but clay pot is rarely used for indoor and outdoor mosquito sampling [9–11]. Although, indoor collection by PSC is used for sampling indoor resting mosquitoes, it interrupts people in the house while spraying in the morning. Window exit traps and resting traps are easy to deploy and do not require a dedicated collector to act as human bait [8, 9]. To understand the blood meal sources and resting sites preference of mosquitoes involved in the transmission of malaria, there is an advantage in collecting mosquitoes using various resting sampling methods. Information gathered through various entomologic collection techniques could contribute to the development and implementation of an effective vector control strategies [12]. Hence, the aim of this study was to assess the composition of the species, the origin of blood meals and the infection rates of resting malaria mosquitoes collected by various resting collection techniques in Aradum, North Wollo, Ethiopia.

## Methods

### Description of the study setting

**Fig. 1:**
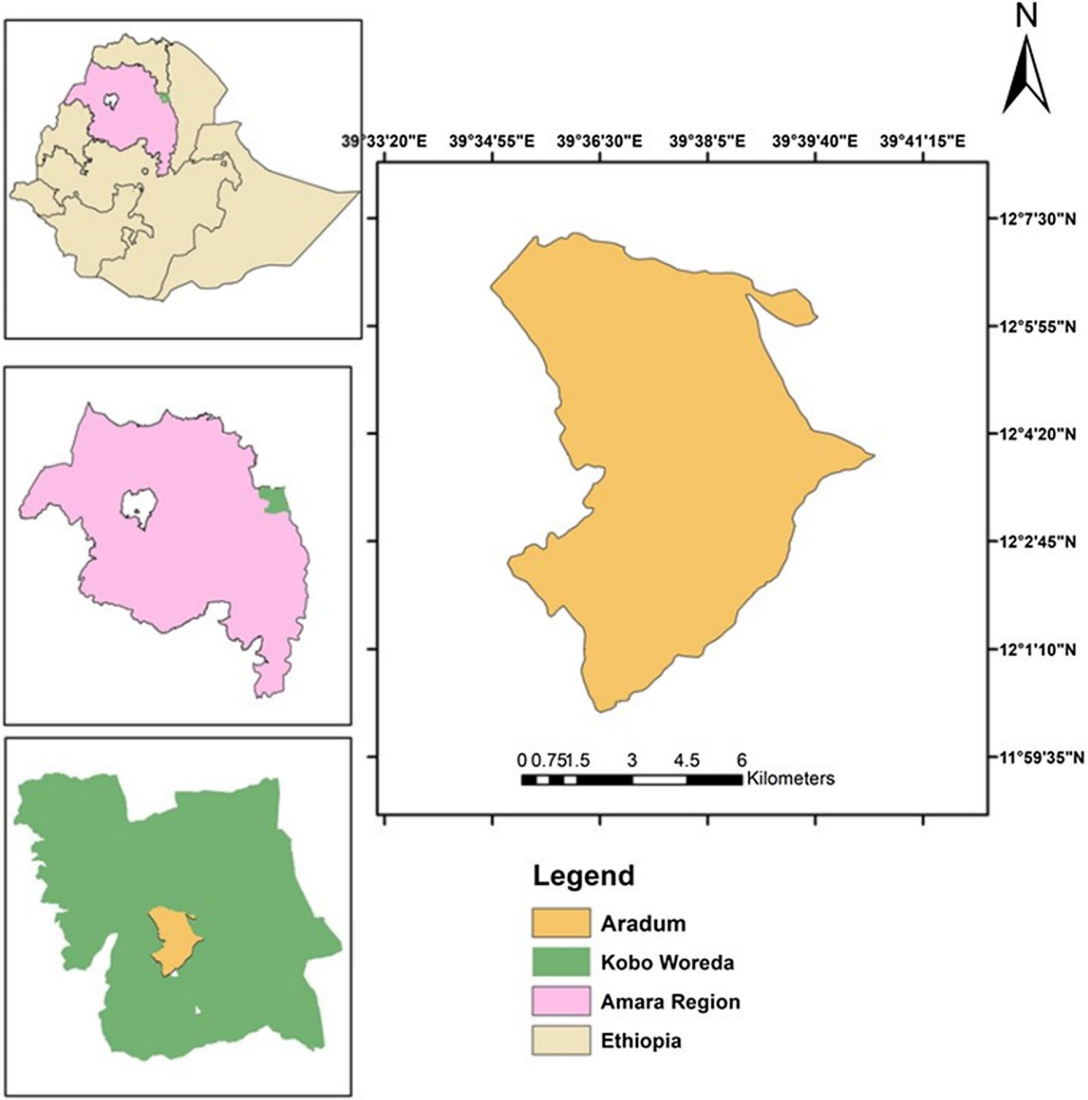
Map of the study area in Kobo Woreda (district), Amhara regional state, Ethiopia (ArcGis 10.4)

The study was carried out in Aradum *kebele* (= village—the smallest administrative unit in Ethiopia) in Raya Kobo district, North Wollo, Amhara regional state, Ethiopia (Fig. 1). Raya Kobo is situated at Latitudes of 11^0^54′ 04" to 12^0^02′ 56" North, Longitude 39^0^25′ 56" to 39^0^ 49′ 04" East. Raya Kobo is rural administrative district with 45 rural villages*,* Aradum is one of these villages. The number of populations in the district is 261,897 (females 128,157; males 133,740) [13]. Kobo town (the centre of the district) is located at 570 km North of Addis Ababa (the capital of Ethiopia), 405 km east of Bahr Dar (the capital city of the regional state), and 50 km from the zonal town, Woldia.

The altitudinal variation of the district ranges between 1260 and 3000 m above sea level (m.a.s.l.). The area is rich in fertile soil and farming is the main mode of living. Sorghum, maize and *teff* are the main cereal crops that the local people produce. The rainy season is unimodal with high rain fall in July and August. The main malaria seasons in the area are July-December [14]. The district has the minimum temperature of 14.1 and maximum 34.1 °C. The mean annual temperature and rain fall reaches up to 27.2 °C and 727 mm, respectively. Water bodies such as rivers and irrigation channels exist in the area. Majority of the inhabitants are farmers and few are merchants, governmental employee and daily labourers. There are 43 clinics, 9 health centers, and one hospital and 3 private clinics. The district has 45 rural villages in which the majority are non-malarious, but Aradum village is among the malarious villages. It is located south of the city of Kobo approximately 5 km away.

## Study design

A longitudinal entomological study was employed from September 2019 to February 2020. The study village was classified into five clusters. A cluster was defined as group of houses closely located on a similar topography. Four houses with rectangular corrugated iron roof and muddy walls were randomly selected from each cluster. A total of 20 houses were included for the study. In each cluster, two houses were randomly assigned for clay pot (indoor and outdoor) and pit shelter (outdoor). The other two houses were assigned for PSC. The distance between the selected houses in each cluster was 500 to 800 m.

## Mosquito collection

### Clay pot collection

Outdoor and indoor mosquito collection using clay pots was carried out twice a month. Four clay pots, two indoor at the foot end of the bed and two outdoor under the shade were used for resting mosquito collection [8, 10]. Each pot was of ≈15–20 litters’ capacity, with an opening of 20 cm width, a round bottom, and a maximum width of 45 cm. Pots were adjusted to have ≈2 cm diameter hole at the bottom to avoiding water holding. Mosquitoes from clay pots were collected in the morning from 6:00–9:00 h. At the time of collection, the opening of clay pots were covered with cloth mesh and the collector then lifted the pot to expose the opening to light and agitate mosquitoes inside, blew into the small hole at the bottom, causing the mosquitoes inside the pot to take flight and enter the cage.

From the cage, they were collected into a labelled paper cup by using handheld mouth aspirator. The cloth mesh was then removed, and remaining mosquitoes in the pot were recovered with an aspirator and transferred into the paper cup, completing the collection. The collected mosquitoes were killed by refrigerating. The abdominal stages of female *Anopheles* mosquitoes were sorted into unfed, fed, half gravid and gravid. Individually, the mosquitoes were preserved in1.5 ml Eppendorf tube and stored in plastic bag containing silica gel for further analysis.

### Pit shelter collection

Outdoor resting mosquitoes were collected from artificial pit shelters (1.5 m of depth, 1.2 m of length and 1 m of width) using handheld mouth aspirator [15]. One pit shelter was constructed at a distance of approximately 8-10 m from selected houses. Inside the four sides of a pit, there were four horizontal holes of about 15 cm wide and 30 cm length. Collection of resting mosquitoes was carried out on the morning from 6:00–9:00 h. While collection, two volunteers stretched white sheet over the pit shelter to prevent mosquitoes from flying away from the pit. Then the collected mosquitoes were transferred into labelled paper cup and into a refrigerator to kill them. Female *Anopheles* mosquitoes were preserved in vials with silica gel.

### Pyrethrum spray collection

Pyrethrum spray collection (PSC) was carried out in the morning from 6:00 to 8:00 h by spraying aerosol (roach killer) manufactured by Kafr El Zayat, Egypt with active ingredients (2% Fenitrothion, Cypermethrin, Bioallethrin, 0.4% Perfume and 97.6% Kerosene and Propellant). This was performed by removing all large pieces of furniture, covering the floor with white plastic sheets. Doors, windows and opening were properly closed. Roach killer was sprayed from outside of the house onto the eaves, windows and door before entering the dwelling and spraying the entire inside of the house. All doors and windows remained closed for 10 min to allow mosquito knockdown. After 10 min, the sheet was brought outside the room and then dead *Anopheles* were collected. The captured *Anopheles* were treated in a similar manner as that of clay pot and pit shelter collection.

### Species identification using morphological key

Morphological species identification of female *Anopheles* was performed using morphological characteristics like palps, wings, abdomen and legs [16].

### ELISA test for blood meal origin and CSPs detection

Female *Anopheles* mosquitoes were tested for *Plasmodium falciparum* and *Plasmodium vivax*_210 CSPs using the Beier et al. [17] procedure and blood meal origin of freshly fed *Anopheles* were identified using ELISA following the procedure of Beier et al. [18] in Arba Minch University Advanced Medical Entomology and Vector Control Laboratory. Head and thorax of females *Anopheles* mosquitoes were used to test for *P. falciparum* and *P. vivax_*210 CSPs, while the abdomen of freshly-fed *Anopheles* mosquitoes was tested for blood meals using anti-human and anti-bovine antibodies.

In brief for CSP test, the monoclonal antibodies were coated on ELISA plate and incubated for half an hour. The plate was aspirated and banged upside down on paper, after which the wells were filled with 200 µl blocking buffer. The samples, negative and positive control were loaded and incubated for 2 h at room temperature. The peroxidase conjugate was added to the wells. Finally, 100 µl of substrate was added. The plate was incubated for 30 min, the colour change was examined visually or with a reader at 405-414 nm.

For blood meal origin test, each mosquito abdomen was crushed in 50 µl phosphate buffered saline solution. Samples were added to each well and incubated overnight at room temperature, then washed twice with PBS containing Tween-20 solution, and host-specific conjugates were added and incubated for one hour. Finally, peroxidase substrate was added to each well and after 30 min the colour change was visualized, and the results was recorded at 405 nm absorbance using an ELISA plate reader.

### Molecular sibling species identification

Sibling species of the *An. gambiae* complexes were distinguished using PCR [19]. The species complexes of *An. funestus* were identified following the method of Koekemoer et al. [20].

### Data analysis

Data were entered and analysed by using IBM^®^ SPSS^®^ Statistics version 20 (Armonk, New York: IBM Corporation). Human blood-meal index (HBI) (proportion of freshly fed *Anopheles* mosquitoes feed on human) and bovine blood-meal index (BBI) (proportion of freshly fed *Anopheles* mosquitoes feed on cattle) were estimated. A nonparametric test (independent-samples median and Kruskal-Wallis tests) was applied to examine the monthly variation and efficiency of the method in terms of capturing the number of *Anopheles* mosquitoes. Test of significance was estimated assuming *P* value less than 0.05.

## Results

### Species composition

A total of 775 female *Anopheles* mosquitoes comprising seven species namely *Anopheles demeilloni, An. funestus* group*, An. gambiae* complex*, Anopheles cinereus, Anopheles coustani, Anopheles longipalpis* and *Anopheles salbaii* were collected using clay pots, PSC and pit shelter. *Anopheles demeilloni* was the predominant species which accounted for 76.5% (593/775) followed by *An. funestus* group (73/775) and *An. gambiae* complex (71/775). The majority (42.2%; 327/775) of the *Anopheles* mosquitoes were collected using pit shelters followed by outdoor clay pots (27.6%; 214/775) and PSC (26.3%; 204/775). Very few *Anopheles* mosquitoes were collected indoor (3.8%; 30/775) by clay pot (Table 1).

**Table 1:** Species composition of *Anopheles* mosquitoes in Aradum using clay pot, PSC and pit shelter North Wollo, Ethiopia

Species composition	Collection methods				Total
	Pit shelter, N (%)	Outdoor clay pot, N (%)	Indoor clay pot, N (%)	PSC, N (%)	
*An. demeilloni*	249 (42)	164 (27.7)	23 (3.9)	157 (26.5)	593
*An. funestus* group	35 (47.9)	17 (23.3)	2 (2.7)	19 (26)	73
*An. gambiae complex*	30 (42.3)	21 (29.6)	2 (2.8)	18 (25.5)	71
*An. longipalpis*	10 (43.5)	7 (30.4)	0	6 (26.1)	23
*An. cinereus*	2 (16.7)	4 (33.3)	3 (25)	3 (25)	12
*An. coustani*	1 (100)	0	0	0	1
*An. salbaii*	0	1 (50)	0	1 (50)	2
Total	327 (42.2)	214 (27.6)	30 (3.8)	204 (26.3)	775

### Monthly distribution of *Anopheles* mosquitoes

**Fig. 2:**
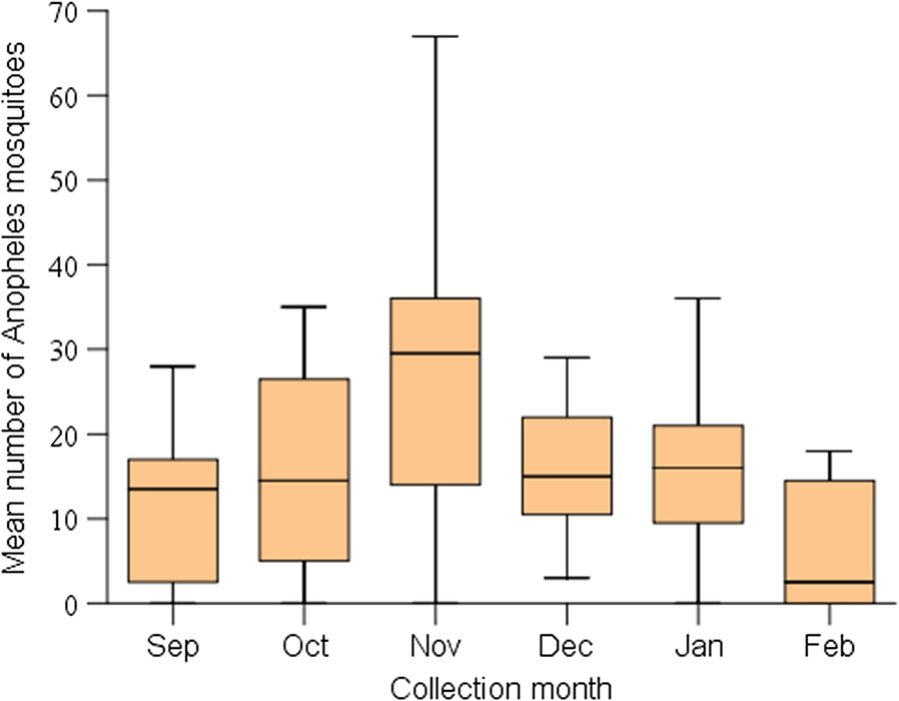
Overall monthly mean number of female *Anopheles* mosquitoes collected by different entomological techniques in Aradum, Northern Ethiopia

The highest number of *Anopheles* mosquitoes (226/775) was collected in November and the lowest number (52/775) was collected in February. The highest mean number of *Anopheles* mosquitoes was recorded in November (28.3) and the lowest in February (6.5) (Fig. 2). The mean monthly distribution of *Anopheles* mosquitoes did not demonstrate a statistically significant difference (χ2 = 9.3; DF = 5; *P* = 0.098). No statistically significant difference was found in the monthly mean number of *Anopheles* mosquitoes collected indoor by PSC, which followed similar trends with a maximum in November and a minimum in February (χ2 = 7.2; DF = 5; *P* = 0.21). The trend was consistent for both the outdoor pit shelter collection (χ2 = 7.9; DF = 5; *P* = 0.16) and the clay pot resting collection (χ2 = 3.5; DF = 5; *P* = 0.63).

### Comparison of *Anopheles* mosquito collection techniques

The outdoor pit shelter was more efficient than other methods in the study site at collecting *Anopheles* mosquitoes (median value: 23.0), but it did not vary significantly from the indoor PSC (median value: 16.5) or outdoor clay pot (median value: 15.5). Compared to other resting collection techniques, the indoor clay pot was the least effective at collecting *Anopheles* mosquitoes (median value: 2.5), and the variation was statistically significant (Table 2).

**Table 2:** The median number of *Anopheles* mosquitoes collected by clay pot, pit shelter and PSC in Aradum, North Wollo, Ethiopia

Collection methods and sites	Test statistics	Sig	Adj. Sig
Indoor clay pot versus outdoor clay pot	16.7	< 0.001	< 0.001
Indoor clay pot versus indoor PSC	10.7	0.001	0.007
Indoor clay pot versus outdoor pit shelter	16.7	< 0.001	< 0.001
Outdoor clay pot versus indoor PSC	0.17	0.68	1.00
Outdoor clay pot versus outdoor pit shelter	0.67	0.41	1.00
Indoor PSC versus outdoor pit shelter	0.67	0.41	1.00

### Blood meal origins

A total of 346 freshly fed *Anopheles* mosquitoes were tested for their blood meal origins. Cattle were the most common blood meal source for the mosquitoes (54.6%; 189/346). Very small proportion of *Anopheles* mosquitoes fed on human (4.6%; 16/346). A substantial number of (37.3%; 129/346) *Anopheles* mosquitoes blood meal origins were not identified using human and bovine antibodies. The overall mixed blood meal origin of *Anopheles* mosquito was 3.5% (12/346).

*Anopheles demeilloni* (58.5%; 161/277), *An. funestus *sensu lato (*s.l.*), (43.5%; 10/23) and *An. gambiae* (33.3%; 14/42) mainly fed on cattle. A small proportion of the blood meal of *An. demeilloni* (3.6%, 10/277), *An. funestus* (13.0%, 3/23) and *An. gambiae* (7.1%, 3/42) originated from human (Table 3).

**Table 3:** Overall blood meal source of *Anopheles* mosquitoes collected by clay pot, pit shelter and PSC in Aradum, North Wollo, Ethiopia

Species	Tested	+ Human N (%)	+ Bovine, N (%)	Mixed, N (%)	Negative, N (%)
*An. demeilloni*	277	10 (3.6)	162 (58.5)	10 (3.6)	95 (34.3)
*An. funestus* group	23	3 (13.0)	10 (43.5)	0	10 (43.5)
*An. gambiae* complex	42	3 (7.1)	14 (33.3)	2 (4.8)	23 (54.8)
*An. longipalpis*	2	0	2 (100)	0 (0)	0 (0)
*An. salbaii*	2	0	1 (50.0)	0	1 (50.0)
Total	346	16 (4.5)	189 (53.0)	12 (4.3)	129 (38.2)

**Fig. 3:**
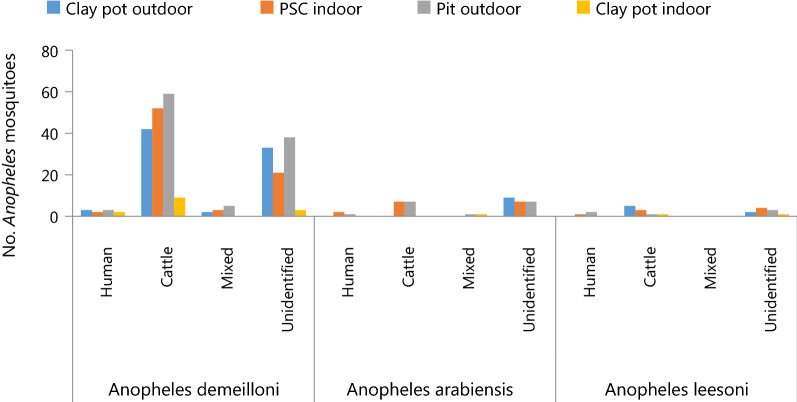
Blood meal sources of *Anopheles* mosquitoes (n = 342) collected by clay pot, pit shelter and PSC in Aradum, North Wollo, Ethiopia

The HBI of *An. gambiae* (presumably *An. arabiensis*) from PSC was 6.2%, while it was 12.5% from pit shelter. The HBI of *An. funestus* (sensibly presumed *Anopheles leesoni*) from PSC was 12.5% and it was 16.6% from pit shelter. However, none of the *An. arabiensis* and *An. leesoni* from indoor and outdoor clay pot traps was positive for human blood meal. Regardless of the resting collection methods, *Anopheles* mosquitoes mainly fed on the bovine blood origins and unidentified blood meal origins mainly observed in clay pot and pit shelter (Fig. 3).

### *Plasmodium *CSPs rates of *Anopheles* mosquitoes

None of 763 *Anopheles* tested for *P. falciparum* and *P. vivax* sporozoite infections were positive.

### Molecular identification of *An. gambiae* and *An. funestus* complexes

Two species of *An. funestus* group were documented by testing 73 specimens by molecular technique. *Anopheles leesoni* was the common (93.2%; 68/73) sibling species, while *Anopheles parensis* accounted only 2.7% (2/73) and the rest 4.1% were not amplified. Of the 71 *An. gambiae* tested for speciation, 91.5% (65/71) were identified as *An. arabiensis* and 8.5% were unamplified.

## Discussion

Morphologically seven species of *Anopheles* mosquitoes comprising *An. demeilloni, An. funestus s.l., An. gambiae* complex*, An. cinereus, An. coustani*, *An. longipalpis* and *An. salbaii* were documented using clay pots, PSC and pit shelter. *Anopheles leesoni* was the predominant species of *An. funestus* complex*. Anopheles arabiensis* was molecularly identified from the *An. gambiae* complex in the region, but there are several specimens not amplified for *An. gambiae* complex. These could be justified by the problem of species misclassification based on morphology or by DNA degradation.

In the present study *An. demeilloni* was the most abundant anopheline species. This is similar with other findings in the region [21]. The dominancy of *An. demeilloni* was recently confirmed in southern highland of Ethiopia [22]. However, in another finding *An. demeilloni* was the least common species documented [10]. *Anopheles funestus* group was the second most abundant *Anopheles* in the study area. Previously, *An. funestus* group had been reported in the region [21, 23]. *Anopheles arabiensis* was another species documented in the study area. Previous study documented that this species was predominant species in Amhara region [10] and other parts of the country [24–26]. The existence of *An. funestus* and *An. gambiae* complexes implies that there should be an attention for proper vector control intervention.

The number of dominant *Anopheles* mosquitoes (*An. demeilloni*, *An. funestus s.l*., and *An.gambiae s.l*.) in the study area was higher after the end of the main rainy month in November. Generally, their number started to increase from September to November but gradually declined from December to February. This finding agrees with Kibret et al. [27] and Adugna et al. [21] who found a higher density of *Anopheles* mosquito between October and November in Ethiopia. An increase in mosquitoes in November may be caused by the availability of favorable breeding habitats at the end of the rainy season. This indicates the necessity of appropriate intervention measures in appropriate timing. For instance, before the vector density in the community reached its peak, the application of IRS and the distribution of ITNs should be taken into account [28]. The control strategy can be implemented at the appropriate time if the seasonal/monthly distribution of the vector population is documented.

The HBI of *An. arabiensis*, *An. leesoni* and *An. demeilloni* from all collection methods was much lower than BBI, indicate a strong zoophilic behaviour of these species in the area. The majority of the blood meals of *An. demeilloni* originated from feeding on cattle, concordantly with what was reported by other studies [10, 29]. Similarly, *An. leesoni* showed zoophilic and an exophilic tendency, which is in harmony with what was documented before [29, 30]. *Anopheles funestus s.l.* contains many sibling species, of which the zoophilic *An. leesoni* was the dominant species documented in the study area. This could provide support for the findings presented in the current study. *Anopheles arabiensis* showed more zoophilic tendency which is consistent with studies in other parts of the country [6, 11, 29, 30]. However, other study reported opportunistic feeding properties of this species [31]. The presence of cattle in close proximity to the households may provide an opportunity to feed on cattle and contribute for the zoophilic behaviour.

The *Anopheles* mosquitoes caught inside the houses by the PSC had much higher proportion of cattle-fed than human-fed, which is less likely to be expected. Normally, the local people keep cattle close to their homes at night outside, and hence mosquitoes might fly into houses to rest after feeding on cattle outdoors. The entire population of *Anopheles* tested was free of *Plasmodium* sporozoite infection, which may have something to do with the mosquito population's feeding habits. This may be related to the diverting effects of cattle toward the dead-end host, which may also help to reduce malaria transmission by wasting their infected sporozoite and lowering the likelihood of infection from human hosts [32]. However, in Ethiopia or elsewhere, *Anopheles* vectors frequently have low or no sporozoite infection rates [11, 33–35]. Interestingly, a substantial number of the origins of *Anopheles* blood meals were not identified using human and bovine antibodies. This may indicate that different blood meal sources exist for malaria mosquitoes as documented in Sudan [36]. As a result, considering all wild animals, including cattle, could have provided a better understanding of the sources of *Anopheles* blood meals in the study area. Additionally, applying insecticides to livestock could be crucial to controlling zoophilic malaria vectors.

Relatively higher proportion of *Anopheles* mosquitoes were collected using pit shelter. The effectiveness of pit shelter over the other resting collection methods is in agreement with other studies [10, 26]. Also, clay pot placed outdoor caught slightly less *Anopheles* compared with pit shelters. This result is comparable with a study done in southwest Ethiopia [11], in which two clay pots were pooled for each pit shelter, showed that a clay pot actually yielded slightly more number of *An. arabiensis* compared to a pit shelter. Unfortunately, high productivity of pit shelter is inconsistency with study in North West Ethiopia [21]. Another study in Addis Zemen south Gondar showed that, only one *An. cinereus* was caught resting in clay pots outdoor [10]. The use of clay pots alone may underestimate the relative density of mosquitoes resting outdoors because mosquitoes may seek alternative resting sites similar to pit shelter. Perhaps using multiple methods to sample mosquitoes resting outdoors can improve understanding of species composition. However, the results of this study showed that clay pots could be an alternative tool for outdoor resting malaria vector surveillance, in settings where using pit shelters is not feasible.

## Conclusion

Of the eight *Anopheles* species documented, *An. demeilloni* was predominant in all indoor and outdoor collection methods. The majority of *Anopheles* blood meals came from cattle, and only a very small percentage of them consumed human blood. A substantial number of *Anopheles* blood meal origins were not identified using human and bovine antibodies. Of the two species of *An. funestus* group documented by molecular technique, *An. leesoni* was the common species. *Anopheles arabiensis* was the only sibling species of *An. gambiae* screened by molecular technique. The clay pot could be an alternative tool for outdoor resting malaria vector surveillance, in settings where using pit shelters is not feasible. Finally, the *Anopheles* mosquitoes in the region tend to feed on cattle and hence animal based intervention could be recommended to target these species.

## Data Availability

All relevant data are within the manuscript.

## Abbreviations

DNA: Deoxyribonucleic acid
ELISA: Enzyme linked immunosorbent assays
HLC: Human landing catch
ICP: Indoor clay pot
IgG: Immune globin G
ITN: Insecticide treated net
OCP: Outdoor clay pot
PCR: Polymerase chain reaction
PSC: Pyrethrum spray collection
HBI: Human blood-meal index
BBI: Bovine blood-meal index
